# Oral health knowledge in relation to educational level in an adult population in Spain

**DOI:** 10.4317/jced.56411

**Published:** 2019-12-01

**Authors:** Cecilia-Fabiana Márquez-Arrico, Jose-Manuel Almerich-Silla, Jose-Maria Montiel-Company

**Affiliations:** 1 Master degree, DDS, pre-doctoral research. Department of Stomatology, University of Valencia, Spain; 2 Lecturer Professor. Department of Stomatology, University of Valencia, Spain

## Abstract

**Background:**

To analyze the relationship between oral health knowledge and educational level among an adult population in Spain, and between oral health knowledge and subjects’ oral hygiene practices, dietary habits, toxic habits, and oral quality of life.

**Material and Methods:**

This transversal study used the Comprehensive Measure of Oral Health Knowledge (CMOHK) questionnaire to evaluate subjects’ knowledge and understanding of oral health, and the World Health Organization oral health questionnaire for adults to evaluate dietary, oral hygiene, toxic habits, and oral quality of life. Participants (n=400) gave their informed consent and data release permission before taking part in the study, which was approved by the University of Valencia Ethics Committee (certificate No.: H145160675341). Statistical analysis was performed using SPSS v22.0 software, applying Student’s t-test, ANOVA, and chi2 test, with significance set at *p*<0.05.

**Results:**

Oral health knowledge results were: low 41.5% and high 58.5%. An association was found between educational level and oral health knowledge (Chi2: *p*=0.000). Oral hygiene habits presenting an association with higher levels of oral health knowledge were dental floss use, a higher number of teeth present, and lower prevalence of partial prostheses. A significant association was found between oral quality of life and oral health knowledge.

**Conclusions:**

Oral health knowledge is associated with the individual subject’s educational level. But oral health knowledge is not necessarily reflected in the practice of healthy habits.

** Key words:**Oral health literacy, oral health habits, oral health knowledge, CMOHK.

## Introduction

Oral health education is a fundamental tool for the prevention of most bucodental diseases. This aims to induce subjects to adopt and maintain healthy habits, make use of the healthcare services available and to make decisions, both individually and collectively, to improve both their oral health and circumstantial factors affecting oral health. Over the last ten years, a number of researchers have developed tools for measuring knowledge and understanding of oral health issues and practices among adults, as awareness is the first step towards a healthy lifestyle. The tools available for measuring oral health knowledge are based on assessments of understanding, knowledge, identification or a combination of these, by means of short questions and/or multiple choice questions about oral health and bucodental hygiene practices (1). Using oral hygiene questionnaires helps to assess the efficacy of healthcare interventions, dental caries prevention campaigns, or anti-smoking campaigns, among others. Although there are many questions concerning oral health habits, the use of a standardized method such as the World Health Organization (WHO) questionnaire, will help to compare and contrast the results and obtain homogeneous data for planning healthcare programs (2).

One of the most widely used questionnaires for measuring oral health literacy in adults is the REALD questionnaire “Rapid Estimate of Adult Literacy in Dentistry” (3). The first version of this questionnaire was created by Richman *et al.* in 2007, based on REALM, a questionnaire created for assessing general medical literacy. The REALD-99 consisted of 99 items with a simple point-scoring method; patients are asked to read lists of oral health terms, awarding one point for every word read and pronounced correctly, obtaining a maximum score of 99 points. This instrument made it possible to compare the scores obtained and analyze their associations with other socio-demographic variables such as age, sex, educational levels, and other variables recorded in the questionnaire. As this is a lengthy questionnaire, it was decided to create a shorter version, without compromising the validity of the original, which led to the REALD-30, launched in 2007 by Lee *et al.* (3).

The REALD and the REALM are valid tools for evaluating oral health literacy, but they do not assess subjects’ real understanding of oral healthcare. Subjects are often familiar with a term, and can pronounce it correctly, but it remains unclear whether he/she really understands its meaning. So, to fill this gap, the ToFHLiD6 (functional understanding of oral health terminology and numerical capability) Test of Functional Health Literacy in Dentistry was developed in 2007 (4). This tool assesses the subject’s understanding and numerical capability, and was the first tool available for evaluating real understanding of oral health matters (1). In 2010, Macek *et al.* created a questionnaire that combined the REALM (oral health literacy) and the ToFHLiD. This tool was named the Comprehensive Measure of Oral Health Knowledge (CMOHK) (5) and was validated by the University of Maryland (USA). The advantage of the questionnaire is that as well as measuring whether a subject is capable of understanding concepts relating to oral health, it also determines whether he/she possesses basic knowledge of different dental pathologies.

It is known that a person’s behavior in terms of daily oral hygiene, nutrition, and toxic habits (such as smoking and drinking alcohol) does not depend on awareness and understanding of oral health issues but is subject to much more complex processes. In addition to knowledge and awareness, for an individual to acquire good oral hygiene habits, he/she must be motivated to adopt good habits, supported by circumstances and resources that make it possible to carry out these practices, among other factors (6). This means that although a subject may have good knowledge and understanding of oral healthcare, this is not necessarily reflected in his/her everyday oral health habits.

At the same time, it is useful to obtain information about the socio-economic levels within a study population, as this is usually an influential variable in the prevalence of bucodental pathology. In 2015, an epidemiological study of oral health in Spain found that dental caries and periodontal disease (among other bucodental characteristics and pathologies recorded in the study) were more prevalent at lower socio-economic levels in both children and adults (7). Transversal studies are able to identify those groups more susceptible to bucodental disease as well as those who have not received sufficient health education.

The main objective of this study was to analyze the relationship between oral health knowledge according to the CMOHK questionnaire, and educational levels, oral hygiene habits, age, and sex in an adult population. It also analyzed the relationship between levels of oral health knowledge and oral quality of life.

## Material and Methods

This transversal study took the form of a survey based on questionnaires, one evaluating oral health knowledge, and another oral hygiene habits, among a population of adult (aged over 18 years) male and female Spanish dental patients and the individuals accompanying them. The survey was conducted at the Dental Clinic at the University of Valencia. Field work was carried out between September 2017 and June 2018 (n=400).

The questionnaires were completed in the waiting rooms at the University of Valencia Dental Clinic, and were filled out by both patients and individuals accompanying them. Each subject was provided with full information about the study objectives and gave their informed consent to take part and data release permission. The study protocol was approved by the University of Valencia Ethics Committee (Certificate no. H145160675341). When the informed consent form had been signed, the subject was given two questionnaires (one to evaluate oral health knowledge and another to assess oral hygiene habits and other variables). When completed, both questionnaires were returned to the surveyor who remained present throughout the process to answer any questions. Each participant was allotted an identification number. Thereafter each participant remained anonymous, as did all personal data such as age, sex, profession, and educational level.

-Questionnaires

The CMOHK questionnaire was used to evaluate subjects’ oral health knowledge (5). This consists of 26 multiple-choice questions with only one correct answer. Questions 1-3 evaluate dental variables, which do not contribute to the oral health knowledge results, but provide other information for further analysis. The CMOHK awards a point for each question answered correctly, making a total maximum score of 23 points, sub-divided into domains corresponding to areas of bucodental knowledge and understanding. The CMOHK questions are grouped as six domains: 1. General dental knowledge (questions 4, 6, 25, and 26); 2. Knowledge about children`s oral health, disease and prevention (questions 5, 7, 13, and 21). 3. Knowledge of oral disease prevention (questions 9, 11, 12, and 16); 4. Knowledge of dental treatments (questions 8, 10, 17, 18, and 20); 5. Knowledge of periodontal disease (questions 19, 22, 23, and 24); 6. Knowledge of oral cancer (questions 14 and 15). Following the method described by Patiño *et al.* in 2015, two levels of knowledge were determined: low level of knowledge (0-14 points), high level of knowledge (15-23 points).

The WHO oral health survey fifth edition (2013) was used to evaluate oral health habits, which also records age, sex, educational level (question 16) as demographic and socio-economic variables. The questionnaire records the number of teeth present, the presence/absence of removable prostheses, and how subjects view their own oral health. Subjects are asked about their oral hygiene practices including the frequency of tooth brushing, use of dental floss, mouthwashes, interspace brushes, etc. Questions 10 and 11 record the frequency of visits to the dentist and the reasons for them. The survey has a section that records 12 oral quality of life items (question 12), and nine items concerning the consumption of sugary foods and drinks (question 12), and toxic habits (alcohol consumption and smoking) (Questions 14 and 15).

To calculate odds ratios (OR) of significant variables, it was necessary to dichotomize oral quality of life variables as two categories. Category 1 grouped responses related to discomfort reported to be very frequent or fairly frequent, while the category 0 grouped responses that reported no discomfort during the last 12 months. To assess the relationship between levels of oral health knowledge and educational level, educational levels were categorized as one of three groups (low, medium or high). A low educational level consisted of subjects who had not received any formal schooling or had not completed primary education; medium included subjects who had completed secondary education or had undergone vocational training; a high level comprised subjects who had completed higher education to degree or post-graduate level.

-Sample size calculation 

It was calculated that a random sample of 389 individuals would be sufficient to estimate, with a 95% confidence interval (CI) and +/- 0.45 units precision, a population mean obtained by the CMOHK of 15.2 points, which was predicted to show a standard deviation of around 4 units. The replacement percentage necessary was predicted to be 20%. To make this calculation, data were used from a pilot study using the same methods applied to a similar population of 171 subjects.

-Statistical analysis

Statistical analysis was performed with SPSS 22.0. Software. For quantitative variables, means and confidence intervals were calculated, while proportions were calculated for categorical variables. Student’s T-test and analysis of variance (ANOVA) were used to compare means. The Chi2 test was used to compare proportions. Statistical significance was set at *p*<0.05. Logistic regression was performed using the forward conditional method with low oral health knowledge as dependent variable and age and educational level as predictive variables.

## Results

-Descriptive results

A total of 439 participants handed in questionnaires, of whom 412 had completed them (a response rate of 93.84%). Twelve subjects were discarded, eight because various options had been marked in multiple choice questions, two because only one of the two questionnaires had been completed, and two because the subjects could not read Spanish. Therefore, a total of 400 subjects were included in analysis. Of the 400 participants, 237 were women (59.3%) and 163 men (40.8%). The mean age of the sample was 45.2 ± 13.7 years. Regarding age groups, 144 participants were aged between 18 and 40 years (36.4%), 172 between 41 and 55 years (43%) and lastly 83 subjects were aged over 55 years (20.8%). Subjects’ educational level was low in 20.0% cases, medium in 38.3%, and high in 41.8%. The mean score obtained in the questionnaire was 14.7 points (IC-95% between 14.3 and 15.1). The scores obtained were classified as two levels of oral health knowledge: low level (<14 points) 41.5% (n=116) and high level (>14 points) 58.5% (n=234).

-CMOHK questionnaire results

The mean score obtained in the CMOHK was significantly higher among young subjects (18-40 years). As age increased the mean score obtained, both in the total score and subdivided scores allotted to the questionnaire’s different domains decreased significantly. When the results were analyzed according to sex, women achieved a significantly higher mean score than men, a pattern repeated in the domains “Children’s oral health, disease and prevention” and “Periodontal Disease.” Educational level also showed a relationship with mean CMOHK scores. Subjects with a high educational level (subjects who had completed higher education to degree or post-graduate level) obtained a significantly higher mean score both in overall scores and in individual domains, compared with subjects with medium and low educational levels ( Table 1).

**Table 1:**
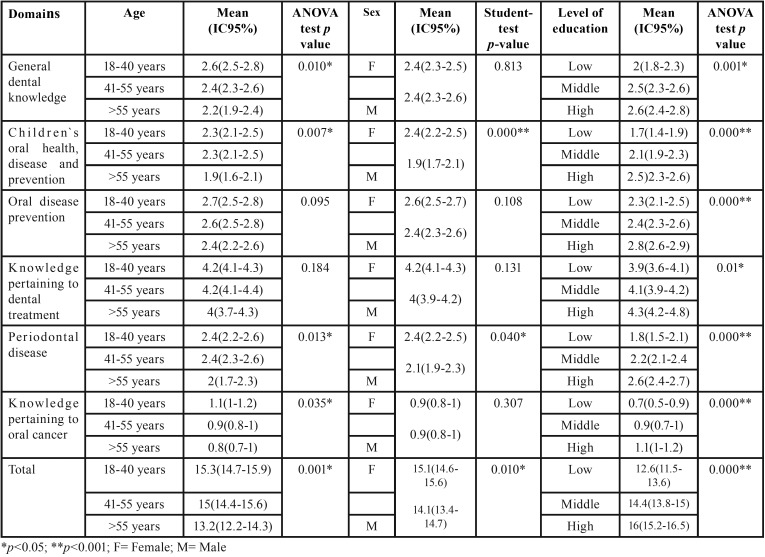
Mean CMOHK scores by domain, in relation to age, sex, and educational level.

When the results were analyzed in terms of oral health knowledge levels, it was seen that subjects who had reached higher educational levels had significantly higher levels of oral health knowledge than subjects with medium or low educational levels. A linear tendency could be observed between the categories (*p*=0.000) whereby as educational level increased, so did the percentage of individuals with a high level of oral health knowledge. The same tendency was observed with the age variable, so that younger subjects (18-40 years) obtained significantly higher average oral health knowledge scores ( Table 2).

**Table 2:**
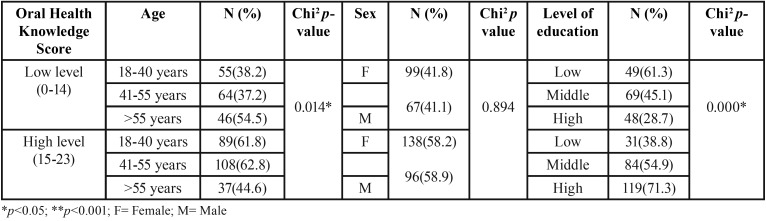
Association between oral health knowledge according to CMOHK and variables age, sex, educational level.

A low educational level was found to be a predictive factor for a low level of oral health knowledge, compared with subjects with medium or high educational levels (OR 1.929). Age was also found to be a predictive factor of a low oral health knowledge level. Older subjects were shown to show almost 2% greater risk of having a low level of oral health knowledge (OR 1.015) ( Table 3).

**Table 3:**
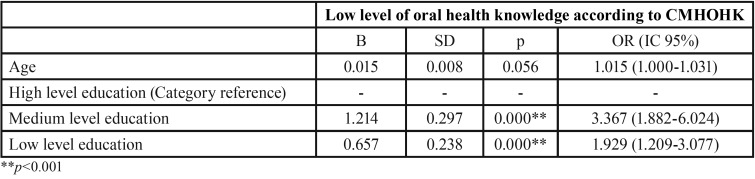
Logistic regression model, with forward conditional method.

-Association between habits and oral health knowledge

Oral hygiene habits that showed an association with a higher level of oral health knowledge were: use of dental floss (49.1% of subjects with a high level of oral health knowledge used dental floss in comparison with 28.9% of subjects with a low level of knowledge [*p*=0.000]); higher number of teeth present (82.9% of subjects with a high level of knowledge also presented 20 or more natural teeth compared with 67.9% of subjects with a low level of knowledge [*p*=0.004]); and lower incidence of removable partial prostheses (22.3% of subjects with a low level of oral health knowledge had a removable partial prosthesis compared with 12.4% among subjects with a high level [*p*=0.009]). Frequency of tooth brushing, the use of mouthwashes and inter-space brushes did not present significant associations with levels of oral health knowledge.

Smoking and alcohol consumption did not present significant associations with levels of oral health knowledge. Nor was any significant association found between the consumption of sugary foods and drinks and oral health knowledge.

-Oral quality of life results in relation to CMHOK oral health knowledge levels

The overall oral quality of life variable was calculated by totaling each of the sections in Question 12 of the WHO oral health questionnaire. According to this instrument, the higher the score obtained, the higher the subject’s level of oral quality of life affectation. To determine the relationship between oral quality of life and oral health knowledge level, Student’s T-test was applied comparing the mean quality of life score obtained by subjects with high and low levels of oral health knowledge. Subjects with low knowledge levels reported “Difficulty in biting foods,” “Difficulty with speech/trouble pronouncing words,” “Felt tense because of problems with teeth or mouth,” “Have avoided smiling because of teeth,” “Have taken days off work,” and/or “difficulty doing usual activities” (during the previous 12 months) with significantly greater frequency than subjects with high levels of oral health knowledge ( Table 4, Table 4 continue).

**Table 4:**
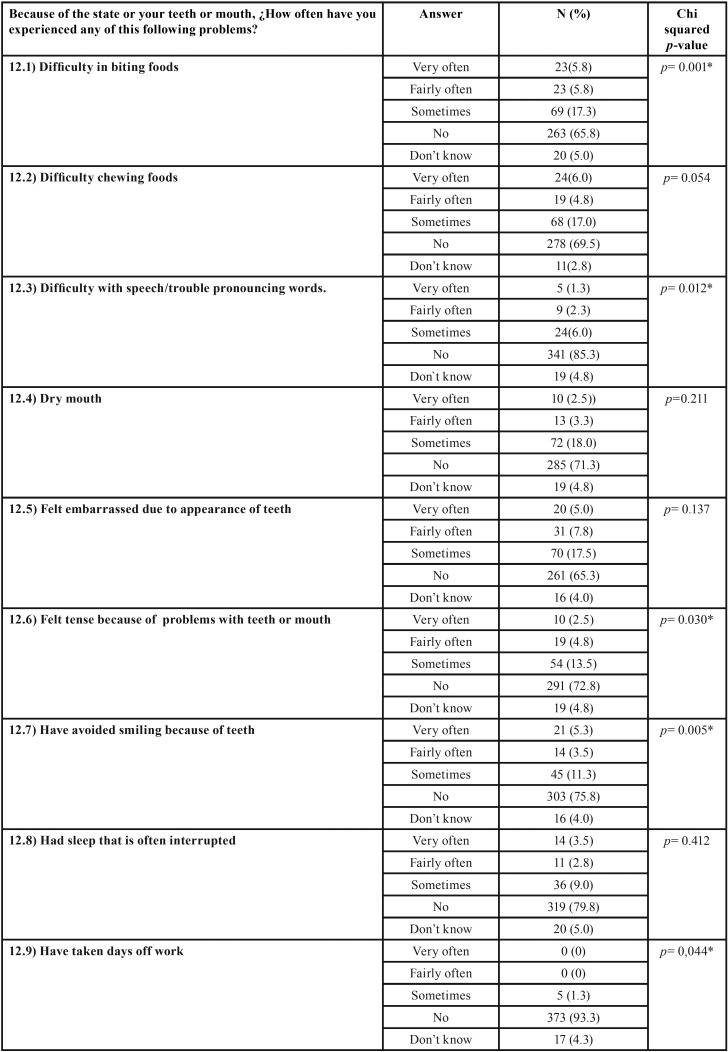
Results of WHO oral quality of life questionnaire. The chi-squared test was applied to analyze association between CMOHK oral health knowledge level and oral quality of life.

**Table 4 continue:**
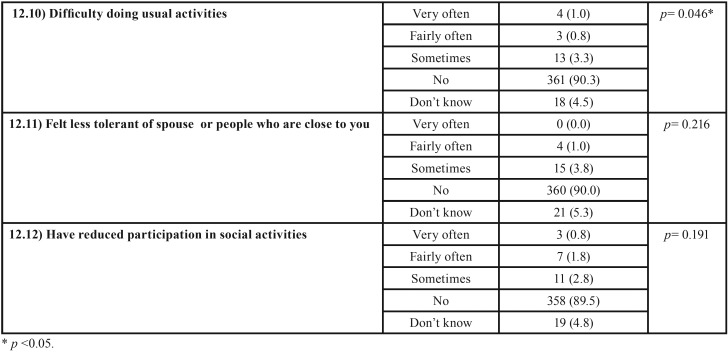
Results of WHO oral quality of life questionnaire. The chi-squared test was applied to analyze association between CMOHK oral health knowledge level and oral quality of life.

## Discussion

The results obtained in the present study concur with those obtained in other research analyzing the relationship between oral health knowledge levels and educational levels. The mean score obtained using the CMOKH was very similar to the score obtained by Patiño in 2015 (around 14 points). Macek *et al.* (2010) and Naghibi *et al.* 2014) (8) found that the domain with the lowest scores was knowledge of oral cancer, while the highest score was for knowledge of dental treatments.

The incidence of cancer of the lips and oral cavity varies according to geographical area and population. Globally, oral cancers represent 3.8% of all cancers. Worldwide, it is estimated that there are 529.500 new cases of oral cancer annually, leading to 292.300 deaths. Cancer of the lip, mouth and throat occupies fourteenth place among all tumors. In Spain, 4,980 new cases of lip, mouth or throat cancer are recorded among men annually, and 1,690 new cases among women (per 100,000 persons per year). Every year, 1,500 Spaniards lose their lives to oral cancer but only 20-30% of oral cancers are diagnosed at early stages (9). Oral cancer is clearly a topic of great relevance and yet unawareness of this oral health issue persists among the general population.

Regarding levels of oral health knowledge in relation to sex, more women were found to present higher levels of knowledge than men. This agrees with other authors (5,10,11) who found that women obtained significantly higher scores in results grouped according to domain. The study by (12) also found that women obtained better results than men although without statistically significant difference.

Investigating educational levels in relation to levels of oral health knowledge, it was observed that as subjects’ educational level increased, so did their level of oral health knowledge, with a linear tendency among the categories. These results coincide with those obtained in other studies, so that subjects who have not entered higher education obtained lower overall scores both in total scores and in scores for individual knowledge domains (10,12-15).

Comparing the present results with data obtained in other countries and other populations, the association between a subject’s oral health knowledge and age, sex and educational level is consistent (12,13,16-18). A study conducted in China in 2019 with a sample of 263 middle-aged subjects found a significant relation between age, low educational level, and low oral health knowledge. Socioeconomic level also influenced oral health knowledge, whereby subjects with less purchasing power showed lower levels of oral health knowledge. Poor oral health knowledge was also associated with deficient oral hygiene and higher numbers of lost teeth. These results show how training in oral health is reflected in a subject’s capacity to maintain oral hygiene and in the consequences, for example, retaining more healthy teeth (16). In agreement with the present study, it has also been shown how subjects who have completed higher education tend to adopt specific oral hygiene practices such as dental flossing (7,12,16,18). A study of 360 middle-aged Slovak subjects found a significant association between higher education and dental floss use. At the same time, women who had completed higher education showed better oral hygiene habits than men. Low socioeconomic level was found to be a risk factor for poor oral health knowledge and deficient oral hygiene habits (13). A similar relationship between socioeconomic level, oral hygiene habits and educational level was also found among a population in south-east Iran (17); this study with a sample of 264 adults, analyzed oral hygiene habits and levels of oral health knowledge. It was found that subjects who did not reach medium or higher educational levels were not well informed or trained in correct oral hygiene maintenance, and were not aware of the importance of regular dental check-ups. It was also observed that women presented higher levels of oral health knowledge than men (17,19).

In light of the above, the data obtained in different studies conducted among different populations in different countries show great consistency in the association between educational level, oral health knowledge, and certain oral health habits. Oral health education programs aimed at groups with lower educational levels may prove useful to bring about a general improvement in oral health.

It is known that quality of life is generally related to socioeconomic level, as purchasing power facilitates access to goods and services, including oral healthcare. A low socio-economic level has been seen to affect oral quality of life (20). In the present study, it was seen how a low level of oral health knowledge did not condition poor oral quality of life, but did influence factors such as pain/discomfort when chewing food, as well as problems in speaking and pronunciation, these being related to early dental loss and the presence of removable partial prostheses, variables that were significantly more frequent among subjects with low levels of oral health knowledge.

The present study’s main limitation was that its transversal design was unable to signify causality. In addition, the sample selection was not random as most of the patients and their companions/family members were attending a dental clinic, a fact that may have compromised the representativity of the sample. Nevertheless, the sample size was adequate and achieved a high response rate (93.8%). Self-completed questionnaires avoided any excessive influence of an interviewer, who was only present to clear up any queries about how to complete the questionnaire correctly.

## Conclusions

As a general conclusion it may be stated that there is an association between the educational level of the adult population studied and levels of oral health knowledge; as the educational level of the subjects increased so did their oral health knowledge. Subjects aged over 55 years presented a lower level of oral health knowledge; as age increased mean scores for oral health knowledge decreased. Women showed a higher level of oral health knowledge than men, particularly in the domains children`s oral health, disease and prevention, and periodontal disease. An association exists between oral quality of life and level of oral health knowledge. Subjects reporting difficulty chewing food, problems with speaking and pronunciation, felt concerned with the state of their teeth, avoided smiling, took time off work or experienced difficulty carry out everyday tasks because of dental discomfort, also showed a significant association with a low level of oral health knowledge.
